# One-session bilateral sequential whole lung lavage (OSBSWLL) for the management of pulmonary alveolar proteinosis

**DOI:** 10.1186/s12890-021-01734-w

**Published:** 2021-11-09

**Authors:** Javier Diaz-Mendoza, Eduardo Celis Valdiviezo, Niral M. Patel, Michael J. Simoff

**Affiliations:** 1 Interventional Pulmonology, Division of Pulmonary and Critical Care Medicine, Henry Ford Hospital, K-17, 2799 W Grand Blvd, Detroit, MI 48202 USA; 2 Department of Medicine, Wayne State University, Detroit, MI USA; 3 Pulmonary and Critical Care Medicine, Moffitt Cancer Center, Tampa, FL USA; 4 Department of Medicine, University of South Florida, Tampa, FL USA; 5 Division of Pulmonary and Critical Care Medicine, University of California Irvine, Irvine, CA USA

## Abstract

**Background:**

Whole Lung Lavage (WLL) has been an important part in the management of Pulmonary Alveolar Proteinosis (PAP) since it improves radiologic and clinical parameters. Bilateral WLL is usually performed in two sessions on different days. Few case reports have described one-session bilateral sequential lung lavage (OSBSWLL), and none have described ambulatory management (same-day discharge).

**Methods:**

Demographic characteristics, physiologic parameters, procedure details and outcomes were retrospectively collected on consecutive patients who underwent OSBSWLL for PAP following an ambulatory protocol stablished in our institution.

**Results:**

A total of 13 patients underwent 30 OSBSWLL (61.5% male; mean age 40). The mean SpO2 was 90% (IQR 9) and 94% (IQR 6), before and after OSBSWLL respectively. In 63.3% of cases, patients were discharged home the same day of procedure. Only in two cases (6.6%), patients required post-procedure prolonged mechanical ventilation (> 4 h) due to persistent hypoxia.

**Conclusions:**

OSBSWLL can be performed with same-day discharge.

## Introduction

Pulmonary Alveolar Proteinosis (PAP) was recognized by Rosen in 1958 [1]. It is a rare entity with a prevalence between 3.2 to 6.7 cases per million [2, 3]. More than 90% of PAP cases occur as primary acquired disorders of unknown etiology [1].

The diagnosis of PAP is based on the clinical suspicion and radiologic findings (bilateral and symmetrical confluent airspace disease [4], and/or “crazy paving” pattern seen on computed tomography [5]. The bronchoalveolar lavage effluent has a turbid and milky appearance due to the presence of foamy macrophages or monocyte-like alveolar macrophages with an increased number of lymphocytes. These changes are associated with a large periodic acid-Shift (PAS) positive acellular background of diffuse eosinophilic bodies [4, 6], which is diagnostic of PAP. The presence of lamellar bodies under electron microscope examination can be used to confirm the diagnosis. These abnormalities translate into an enormous accumulation of surfactant lipoprotein in pulmonary alveoli leading to the clinical findings of increase alveolar-arterial oxygen gradient and hypoxemia.

Whole Lung Lavage (WLL), first described by Ramirez-Rivera in 1965 [7], is currently the first line therapy for PAP. This procedure is performed to remove the excess surfactant proteins and lipids from the alveolar spaces in order to improve gas exchange. Most published case series, as well as a recent global survey, have shown that most centers perform WLL on two separate sessions [8]. In our institution, we have been performing bilateral, sequential WLL on one session (OSBSWLL) since 1994, although inconsistently initially, it became more consistent over the past 15 years. We believe that OSBSWLL is safe to perform, comfortable for the patient, and could reduce costs of a second procedural session. Per our knowledge, this is the largest case series of OSBSWLL reported.

## Methods

We retrospectively reviewed the medical records of patients who underwent WLL due to PAP from 1994 until 2013 at Henry Ford Hospital. Our Institutional Review Committee (IRB) had approved this study (IRB-9021) in accordance with the relevant guidelines and regulations. A total of 25 consecutive patients who underwent WLL were identified. All patients had biopsy proven PAP. 13 patients underwent a total of 30 OSBSWLL. Data regarding demographics, clinical presentation, procedure, and complications were collected (Table 1).

**Table 1:** General demographics

Patient	Age	Gender	Race	Diagnosis	Year of diagnosis	Years to first OSBSWLL
1	53	M	AA	BAL TBBX	1996	4
2	64	M	C	BAL TBBX	1996	< 1
3	29	F	AA	BAL TBBX	2000	< 1
4	35	M	H	OLBX	2000	< 1
5	29	F	C	OLBX	2012	< 1
6	50	M	C	OLBX	1997	1
7	16	M	C	BAL TBBX	2000	< 1
8	44	M	C	OLBX	2007	< 1
9	46	F	C	OLBX	2007	2
10	46	F	AA	OLBX	2009	2
11	32	M	C	OLBX	1996	< 1
12	40	F	C	BAL TBBX	1995	< 1
13	44	M	C	OLBX	1994	< 1

AA: African American; C: Caucasian; H: Hispanic; OSBSWLL: one-session-bilateral-sequential whole lung lavage; OL BX: open lung biopsy; BAL TBBX: bronchoalveolar lavage and transbronchial lung biopsy

### Procedure/technique

#### Before lavage

**Fig. 1:**
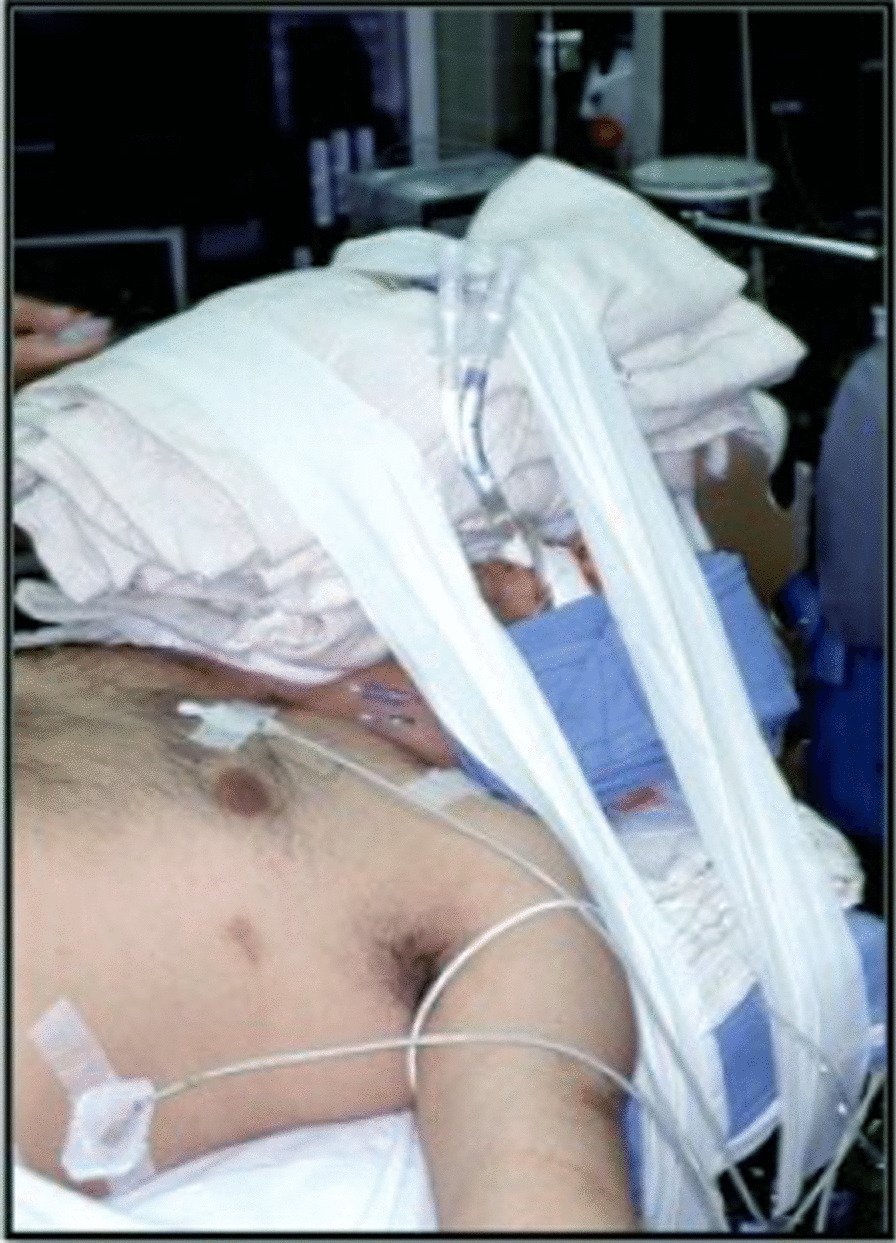
Patient intubated with a double lumen endotracheal tube supported by towels achieving airway stabilization and protection of patient soft tissue areas over the neck, face and head

All OSBSWLL are performed in the Operating Room (OR) under general anesthesia. The team involved in the procedure include: an interventional pulmonologist, an anesthesiologist, respiratory therapists, and experienced operating room assistants. In the operating room, general anesthesia and muscle paralysis are induced by the anesthesiologist. The patient is placed in a supine position and intubated with a 39 Fr double-lumen endotracheal tube (dETT) (or smaller depending on the size of patient). After the position of the dETT in the airways is confirmed by flexible bronchoscopy (pediatric scope), the dETT is secured in place. A “leak test” is then performed: a single lumen endotracheal tube is attached to a connector allowing it to be secured to the dETT, with the tip placed in a bottle of saline, which will hence be referred to as the leak test kit (LTK). Initially one side of the dETT is connected to the LTK and the other to the ventilator. Airway pressures are increased by the ventilator and held sequentially at 20, 30 and 40 cm H2O. The airway pressures are then fluctuated between 40 and 50 cm H2O looking for bubbling in the saline from the end of the LTK. If no bubbling is observed, the process is repeated on the opposite side of the dETT. If both “leak tests” are negative, we proceed to stabilize the head of patient and tubing system by using towels, blankets and padding to avoid pressure ulcers on the patient’s face and skin (Fig. 1). The flexible bronchoscope is then used to confirm positioning one more time.

**Fig. 2:**
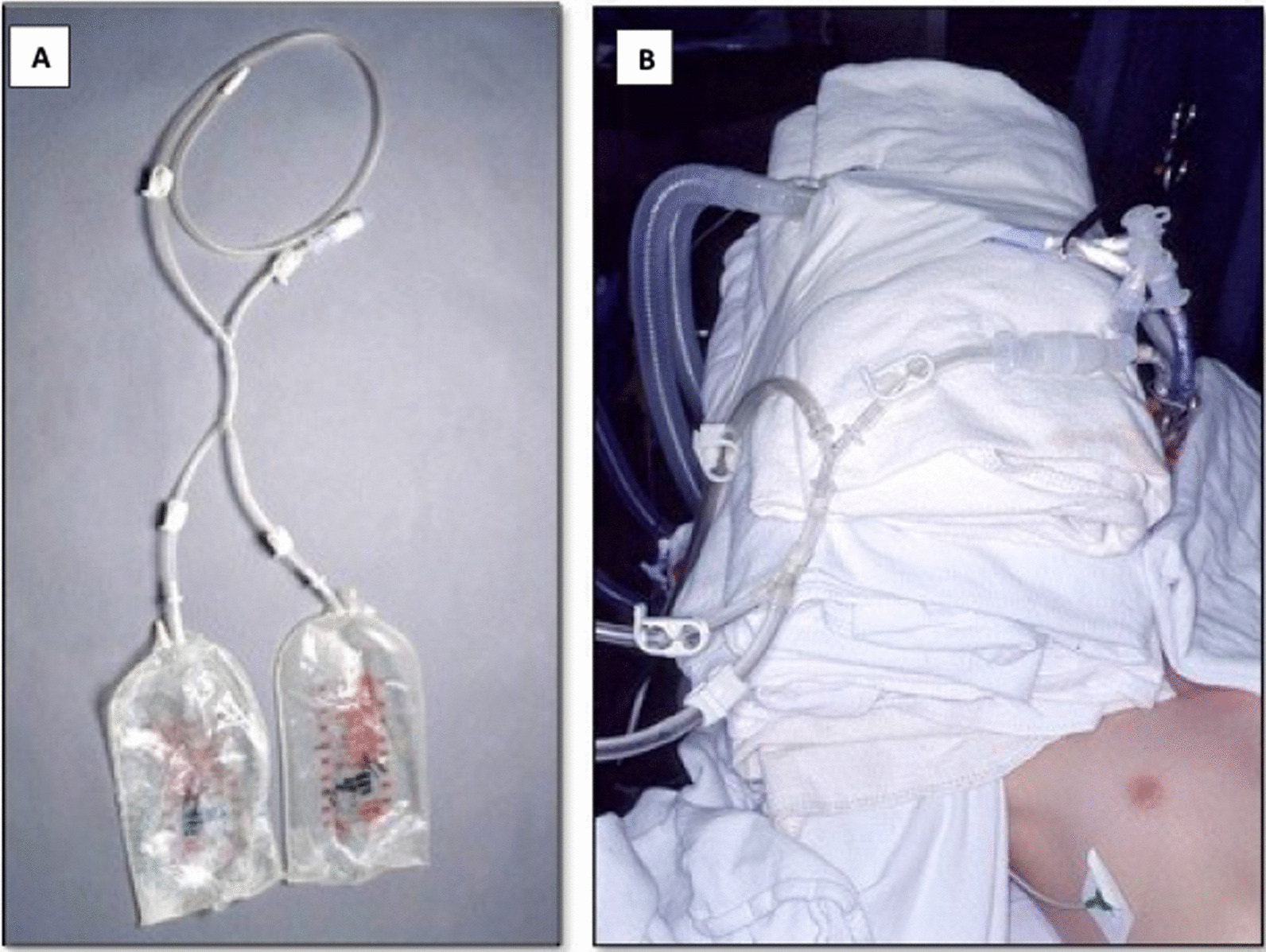
**A** Tubing setting. **B** Tubing setting seen on patient during procedure

The patient is then oxygenated with 100% FiO2 to both lungs for 10 min. After the first lung to be lavaged is determined (the lung with the greater disease burden by imaging), we proceed to the “degassing maneuver”. For this, the corresponding arm of the dETT is closed using a curved Kelly clamp. The dETT remains clamped for 5 min to allow degassing of the lung. [9] The customized tubing system to be used for the lavage (Fig. 2) is prepared, ensuring that it is air-free. It is then connected to the appropriate arm of the dETT. Multiple three-liter bags of normal saline had been previously warmed to a temperature of 37 °C. The bags are then hung 40 cm above the level of the heart.

#### Lavage

**Fig. 3:**
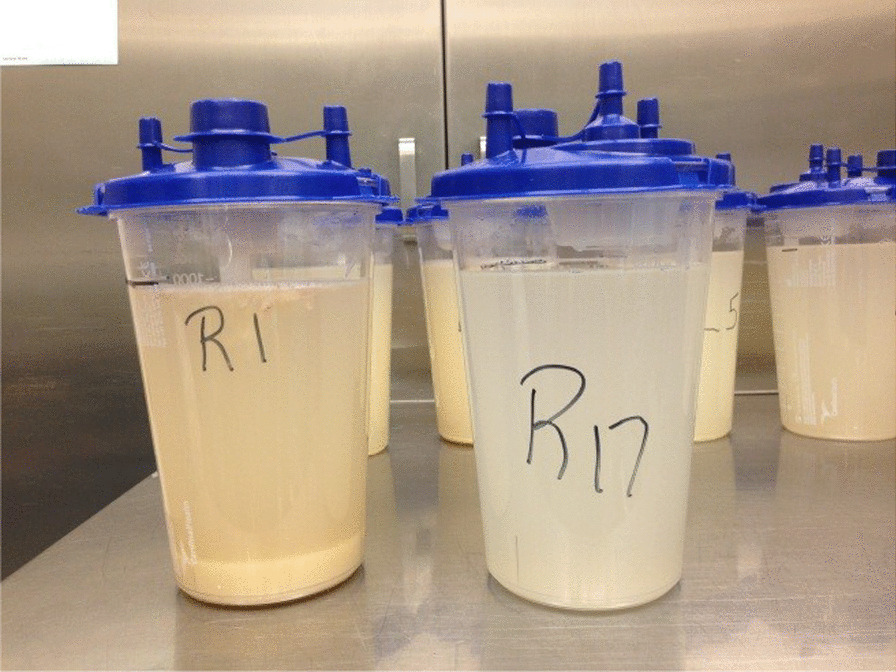
Example of visual assessment of returned lavage. R1: first returned lavage, right lung. R17: 17th returned lavage, right lung

We start the lavage by instilling two liters of the warmed normal saline, by gravity, into the lung through the tubing system. Cardiopulmonary physical therapists provide manual percussion of the chest throughout the procedure in order to achieve better mobilization and subsequently improved removal of particulate matter and instilled fluid. After the first two liters are instilled into the lung, the inflow portion of the tubing circuit is closed and the effluent from the lung is drained by gravity into a suction canister which is calibrated by milliliters. Initially, one liter of effluent is drained and one liter of saline remains in the lung to maintain patency of the lung. The process of instillation and drainage is repeated using one-liter aliquots for both the in and out portions of the procedure. Visible clearing of the effluent is used as the marker to stop the lavage. Effluent clearing usually requires approximately 16 L of fluid per lung. When the effluent has cleared enough (Fig. 3), the final drainage will include the one liter of initially instilled into the lung.

The patient is then reassessed to decide if the procedure for the second lung can safely be completed. Assessment of vital signs, including pulsoximetry, as well as bronchoscopic confirmation of the dETT are done before moving on to the second lung. Then we proceed to perform the “degassing maneuver” on the second lung (as described above).

The amount of fluid instilled and removed is carefully ascertained at the completion of each lung and then for a total for the procedure. The “final balance of fluid” (total amount of fluid instilled into the lungs minus amount of fluid recovered from the lungs) is recorded.

#### After lavage

At procedure terminus, the double-lumen tube is removed and a #8 single-lumen ETT is placed (depending on the size of patient). The patient is maintained on mechanical ventilation for 4 h following the procedure. For the first 2 h on an FiO2 of 100% and for the second 2 h weaned to 30%. If patients are assessed to be clinically doing well with adequate SaO2 they are discharged home the same day of the procedure. Patients admitted to the hospital overnight will be weaned to room air prior to discharge. All patients are scheduled for outpatient follow up within 1 to 2 weeks.

## Results

Thirteen patients underwent a total of 30 OSBSWLL (mean of 2.3 OSBSWLL per patient, IQR 1). There were two other procedures scheduled initially as OSBSWLL, but they only completed a single WLL due to complications. These 2 cases were not included on the 30 OSBSWLL (Table 2).

**Table 2:** Procedures scheduled as OSBSWLL, but only completed lavage of one lung

Patient	Reason for stopping procedure	Admission to hospital	ICU days	Total hospital days
10	Pneumothorax, needed chest tube placement	yes	3	5
12	Hemodynamically instability, that resolved spontaneously	no	NA	NA

Eight patients were male (61.5%). Nine were Caucasians (69.2%). PAP was diagnosed with Open Lung Biopsy (OLBX) in 8 patients (61.5%). The time from diagnosis of PAP to first OSBSWLL ranged from less than one year to 4 years (mean 1.38; IQR 0.5) (Table 1).

None of the patients were on mechanical ventilation before OSBSWLL, and only two were inpatients before the procedure (6.6%).

The time used for OSBSWLL was found in 17 procedures. The time varied between 135 and 274 min (mean of 208.1 min, IQR 42). The total amount of normal saline (in Liters) used per OSBSWLL ranged between 14.5 and 50.9 (mean of 31.1, IQR 15.3) with a range between 6 to 29.3 per lung (mean of 15.5 L). The final balance of fluid at the end of the procedure ranged between 0 and 2500 ml (mean of 781.8 ml, IQR 790).

Peripheral capillary oxygen saturation (SpO2) values on room air were found in 21 cases (15 pre-procedure, 19 post-procedure, and 13 in both). The mean SpO2 pre-procedure was 90% (IQR 9). The mean SpO2 in the immediate post-procedure was 94.8% (IQR 6). Among the cases with both values, the mean variation between the pre and post-procedure SpO2 was + 5.3% (IQR 9.5).

Eleven cases of OSBSWLL (36.6%) required post-procedure admission to the hospital. The most common cause for admission was observation for dyspnea (Table 3). The mean length of admission was 2.3 days (IQR 1). Only in 3 cases of OSBSWLL patients required admission to the Intensive Care Unit (two cases with 1 day and one case with 7 days) (Table 3).

**Table 3:** Procedure, indication and hospital admission per patient

Patient	Reason for OSBSWLL	SpO2 pre/post lavage at rest on Room air (%)	NS used Left lung (L)	NS used Right lung (L)	Residual volume (mL)	Length of procedure (min)	Reason for admission	ICU days	Total hospital days
1	WD, WI	–/–	17	15	300	–	NA	0	0
2	WD, WI	–/80	8	6.5	2500	–	Hypoxia, pneumonia	7	13
3	WD, WI	–/–	17	15	415	–	Observation (dyspnea)	0	3
3	WD, WI	–/–	27	18	0	–	NA	0	0
4	WD, WI	–/84	17	6	1000	–	Hypoxia	1	1
4	WD, WI	–/–	10.2	9.7	1020	–	Observation (dyspnea)	0	1
5	WD, WI	95/98	29.3	21.6	1025	256	Tachycardia	0	1
5	WD, WI	92/92	21	18	250	219	Observation (dyspnea)	0	1
6	WD, WI	–/–	9	15	1000	–	NA	0	0
7	WD, WI	–/–	18	15	320	–	NA	0	0
8	WD, WI	96/90	21	21	325	135	Observation (dyspnea)	0	1
8	WD, WI	94/98	15	21	1090	207	Observation (dyspnea)	1	1
8	WD, WI	94/96	18	21	2100	206	Observation (dyspnea)	0	1
9	WD, WI	95/94	19.1	24	80	234	NA	0	0
9	WD, WI	92/97	17.8	30	200	268	NA	0	0
10	WD, WI	91/97	21	28	1925	274	Observation (dyspnea)	0	2
11	WD, WI	–/–	21	17.5	200	–	Observation (dyspnea)	0	1
11	WD, WI	–/–	12	12	500	–	NA	0	0
12	WD, WI	–/100	12	13	830	195	NA	0	0
12	WD, WI	–/98	15	15	1050	170	NA	0	0
12	WD, WI	89/97	9	12	1350	200	NA	0	0
12	WD, WI	–/98	11	12.7	0	185	NA	0	0
12	WD, WI	84/100	12	18	550	170	NA	0	0
12	WD, WI	85/100	12	15	500	220	NA	0	0
12	WD, WI	83/96	12	15	500	185	NA	0	0
12	WD, WI	86/90	15	15	0	200	NA	0	0
12	WD, WI	84/–	15	15	850	–	NA	0	0
12	WD, WI	–/–	9	12	1300	–	NA	0	0
12	WD, WI	90/–	9	9	1100	–	NA	0	0
13	WD, WI	–/97	9	9	1175	215	NA	0	0

WD: worsening dyspnea (subjective to patient); WI: worsening chest X-Ray or chest computed tomography images; NA: non applicable; NS: normal saline; L: liters; mL: milliliters; –: no value found

In 19 cases of OSBSWLL (63.3%), the patients were discharged home the same day of procedure. About 1-year survival, 24 cases (80%) of OSBSWLL were alive after 1 year, 1 had died and 5 were unknown.

## Discussion

It is known that WLL is the standard treatment for PAP, since it has a great efficacy to improve symptoms as well as other functional and radiological parameters [1]. There is still a lack of standardization of the indications for the procedure, the procedure itself and the outcomes [8].

Traditionally, most case series of PAP have mentioned the use of WLL in two separate sessions (one lung per session) [3, 4, 6, 8]. A global survey on WLL done in 20 centers in 14 countries revealed that most centers perform WLL in consecutive sessions (one lung per session), with an interval of 1 to 2 weeks between procedures [8]. No specific data has been published about OSBSWLL thus far.

In this case-series, we present our experience with OSBSWLL as part of the treatment for PAP. We believe this is the largest case-series report for OSBSWLL in the literature.

The most common indications to perform WLL described are: lung function decline, change in PaO2 or SpO2, worsening chest imaging (chest X-ray or computed tomography) and worsening dyspnea [3, 4, 8]. In our case-series, all the patients had dyspnea and worsening chest images before undergoing OSBSWLL. The mean SpO2 value before procedure was also low (90%).

The amount of fluid used per lung in our series has a great variation (6 to 29.3 Liters), which is comparable to other series (range between 1 and 40 Liters) [1, 8]. In our institution the lavage continues until the pulmonologist does a visual assessment of the color and sediment of the effluent demonstrating enough clarity to see the movement of fingers through the effluent (Fig. 3). Even though Bonella, et al. [10] used optical density monitoring to quantify protein content of the returned fluid as a tool on the decision to terminate the procedure, there is no data that compares optical density values against visual assessment of color and sediment done by an experienced physician.

The length of time to perform OSBSWLL ranged between 135 and 274 min in our case-series. The duration of single WLL reported by Campo et al. [8] ranged between 120 and 360 min. These results show very similar times between OSBSWLL and single WLL, which could be explained by the fact that the longest part of the procedure is dedicated to positioning the patient and securing/stabilizing the airway, which is the same in both procedures.

The post-procedure management in our cases is similar to other series. Abdelmalak, et al. [11] described an early weaning from the ventilator (within 4 h of finishing the procedure) and weaning of supplemental oxygen. In our series, 63.3% of cases were able to be weaned from ventilator and discharged home the same day of the procedure. In fact, SpO2 values improved by 5.3 points after the procedure; and all patients had a subjective improvement of dyspnea.

Complications reported in other series of WLL have included hypoxemia (which is usually transient, during the emptying of the lung being lavaged, that releases compression of the capillary bed, creating a shunt). Refractory hypoxemia and respiratory failure have been described, mostly related to lavage fluid absorption leading to pulmonary edema [11]. In our series, only 2 cases (6.6%) required mechanical ventilation longer than 4 h after OSBSWLL due to hypoxia. One of them, who was treated for pneumonia post-operatively, also had a large residual volume (2,500 mL) that could have contributed to hypoxia. Pneumothorax has also been described in WLL due to excessive alveolar distention [11]. Even though, none of the patients who underwent OSBSWLL developed pneumothorax, one of the patients who was initially scheduled for OSBSWLL could not complete lavage of the second lung due to hypoxia that was later deemed to be secondary to pneumothorax on the lavaged lung, which required a chest tube placement and admission to ICU. One patient died within 1-year post OSBSWLL, unfortunately no records were able to be collected in terms of cause of death.

We believe that OSBSWLL requires an experienced multidisciplinary team. Planning and communication with the members of the team are the key to the success of this procedure.

Although patients were captured from a prospective, IRB approved database, much of the data was extracted retrospectively. This limited the obtention of some data: clinical presentation, functional outcomes, time of procedure on all patients, overall because most data were recorded before electronic medical records were instituted. Furthermore, despite developing a protocol in our institution, some inconsistency was noted when OSBSWLL started to be performed. Despite all these limitations, we believe that our data can be used to improve future protocols for performing whole lung lavage.

## Conclusions

OSBSWLL can be performed withinone procedure with a same-day discharge as part of the treatment for patients with diagnosis of PAP. OSBSWLL could be considered as part of the management on patients with PAP that require WLL in institutions with a stablished protocol. Standardization of procedural protocols should be developed among institutions.

## Data Availability

All data generated and analysed in this study are included in this article under related information files.
